# Understanding the Microfoundations of Government–Civil Society Relations

**DOI:** 10.1007/s11266-020-00221-w

**Published:** 2020-04-13

**Authors:** Maikel Waardenburg

**Affiliations:** School of Governance, Utrecht University, Bijlhouwerstraat 6, 3511 ZC Utrecht, The Netherlands

**Keywords:** Government–civil society relations, Institutional work, Agency, Service provision, Managerialism

## Abstract

This article adds a much needed microlevel perspective to the literature on interactions between civil society organizations and governments. I argue that a microlevel perspective assists in making connections between two dominant streams in the literature on government–CSO relations: an empirical–analytical stream and a critical stream. It aims to better understand the interactions and relations, by analysing the institutional work done by CSOs’ members. Adopting this approach puts CSO members in a more agentic position. Interactional processes are brought to the centre of analysis. The Dutch Community Sport Coach programme was used as a case to illustrate the usefulness of the approach. Through a one-year organizational ethnography, the article scrutinizes the way in which members of one CSO enact the organization’s service delivery relationship with a municipality. Through a multidimensional perspective on agency, the analysis shows how individual CSO members act as embedded agents that assimilate a public logic into the dominant community logic. It further shows the CSO’s members efforts and struggle to maintain their community logic. The article argues that an analysis of the microfoundations of government–civil society organization relations foregrounds the multivocality of the relationship as foundational.

## Introduction

Civil society organizations (CSOs) are valued and used by governments for contributing to a diversity of public issues. For instance, during the 2020 Corona virus outbreak the Salvation Army in the UK closely worked in partnership with government to serve the most vulnerable people in this time of global uncertainty. In another turmoil situation, Danish municipalities turned to voluntary organizations for refugee reception (Fehsenfeld and Levinsen 2019). Also in less dramatic, more structural issues, governments closely cooperate with CSOs. For instance, Van de Bovenkamp and Trappenburg (2011) showed that Dutch patient organizations are used by the state as a counterforce against health insurers and the pharmaceutical industry in health-care decision making.

This phenomenon of government–CSO interaction has been approached from two streams of research. In the first, empirical–analytical stream of research, terms such as *service provision* and *co*-*production* turn the attention to the service value of the relationship (Bovaird and Loeffler 2012; Brandsen and Pestoff 2006; Evers 2005; Osborne et al. 2016). Articles in this stream typically analyse one or more specific services that have been outsourced by government to CSOs or are produced through investments made by both government and civil society. In this stream of research, government–civil society relations are a label to describe and analyse alternative models of state and CSO interactions (Haddad 2017; Najam 2000; Young 2000).

A second stream of research starts from a critical theoretical position arguing that contributing to government’s services is quickly becoming an institutional norm to which CSOs and their members have to conform (Brandsen et al. 2017; Ilcan and Basok 2004; Mitlin 2008). In the eyes of governmental agencies, organizing for public value and contributing to the revitalization of society seem to be what constitutes CSOs as a virtuous organization (Brandsen et al. 2017). In another vain, the relationship with CSOs might actually be used to re-enforce the power monopoly of authoritarian regimes (Ljubownikow and Crotty 2017; Skokova et al. 2018).

While the interaction between state and CSOs has been widely described from these two streams of research, and multiple organizational consequences for CSOs have been identified (Aimers and Walker 2016; Froelich 1999; Verschuere and De Corte 2014), research in this field is ‘in need of more relevant metaphors, concepts, conceptual relations, and theoretical frameworks that can help us to better understand what occurs on the ground’ (Hvenmark 2016, p. 2835). Although Hvenmark’s remark is specifically directed to the concept of managerialism, I argue this also holds true for the broader debate on government–civil society interactions. In addition, there is a strong call for more attention for the internal microprocesses how organizational members of CSOs enact their relationship with government (Fehsenfeld and Levinsen 2019; Pache and Santos 2013). With this article, I aim to contribute to the ongoing debate on state–CSO interactions by focusing on the institutional work conducted by individual agents.

A better understanding of the microfoundations of interactions between agents in CSOs and their governmental counterparts is important for several reasons. First, state–civil society interactions have meaningful consequences for CSOs members’ identity (Chen 2009; Neilson 2009; Kreutzer and Jäger 2011). Members in mutual support organizations seem to be encouraged to take responsibility for a broader, non-member audience (Ilcan and Basok 2004; Waardenburg and van de Bovenkamp 2014). Yet, the initial commitment of most volunteers is oriented internally, to the CSO’s own members. In addition, members of mutual support organizations could well see more value in their original community-driven activities, rather than new service delivery responsibilities. How do they align these diverging values and identities? Second, state–civil society cooperation might constitute an organizational paradox (Vangen 2017; Miron-Spektor et al. 2018). For instance, the primary existence of many social movements is an oppositional stance towards state agencies. NGOs position themselves often as agents that challenge the state, pressing for social, environmental, or political change (Mercer 2002; Neilson 2009). How do NGO representatives uphold constructive microlevel relationships with state representatives, while simultaneously pressing for governmental change? Third, the literature on government–CSO interactions has predominantly put governments on the dominant side of the relationship and consequently focusing on the negative impact the out-of-balance relationship has on CSOs (e.g. Froelich 1999; Geoghegan and Powell 2006; Skokova et al. 2018). An institutional work approach adopts an agentic perspective, providing room for the analyses of CSOs individual representatives’ influence on and power position in the relationship.

This article zooms in on voluntary sports clubs (VSCs) as one type of CSO. VSCs typically constitute one of the largest civil society sectors in several countries. Governments in countries such as Australia, Germany, the UK, and the Netherlands ascribe VSCs a valuable role in service delivery (Harris et al. 2009; Waardenburg and Nagel 2019). In the Netherlands, as well as in other North West European countries, local governments increasingly use VSCs as partners for contributing to public issues, like counteracting children’s overweight and obesity, promoting social integration of newcomers or people with disabilities, improving social cohesion in neighbourhoods, activating the elderly and developing basic skills of democracy (Albrecht et al. 2019; Ibsen and Levinsen 2019; Jaitner 2019; Waardenburg 2016). Other types of CSOs are dealing with comparable service provision activities. A difference with other CSOs is that VSC’s members are primarily focused on competitiveness (Harris et al. 2009; Stenling and Fahlén 2009; Skille 2011). This field specific logic is not necessarily in line with aims focussing on the creation of public values and the *raison d’être* behind government outsourcing.

In this article, the way in which one VSC in the Dutch municipality of Utrecht enacts its relationship with the municipal government is studied in depth. By adopting the institutional work approach, the article aims to explore and describe government–CSO interactions from a microperspective. The following research question guides the analysis: How do individual actors in a voluntary sport club enact their service delivery relationship with government as institutional work?

This article continues with a further explanation of the institutional work approach and discusses its usefulness for government–civil society interactions. Next, a three-dimensional analytic framework on institutional work is presented. After explaining the methods used, the paper continues with a detailed analysis of an ethnographic case study. I describe how members of one CSO interpreted their interactions with local government. Members understand these interactions through two differing interpretations on professionalization. These lenses of professionalization are enacted to maintain a community logic. The article finalizes with a discussion of the findings.

### Government–Civil Society Organisations Interactions

From the 1980s, Western governments began to realize that they could not tackle the social challenges they faced independently. Societal problems were too complex and governments’ steering power too weak. The traditional view, in which the government is regarded as a controlling and regulatory organization that enables the functioning of society, increasingly appeared to be outdated (Peters and Pierre 1998). New Public Management arrived as the dominant praxis in administrative behavior. This implied that social welfare regimes gradually released their hierarchical role in policy fields from the 1980s onward to make way for a more market-oriented government. This can be seen, inter alia, in the privatization of large state enterprises and, for example, involving CSOs in policy formation and implementation. Later, a network approach in which public and private parties cooperate on societal challenges became the dominant modus operandi (Osborne 2010; Pestoff et al. 2012). These changes resulted in an increase in cooperative relationships between government actors and CSOs, which is expressed in terms of outsourcing, service provision, and co-production (Osborne et al. 2016).

The associated change in orientations within associations is not without consequences. Brandsen et al. (2014) found that cooperation between civil society and government in providing public services has led for many CSOs to a loss of contact with the traditional membership base and, consequently, their original legitimacy. CSOs gain that legitimacy, especially from below and from the inside, from its association members. But as an executive of public duties, associations might derive their legitimacy increasingly from the above and outside of the organization. Consequences are diverse. For patient associations, Van de Bovenkamp and Trappenburg (2011) found that their ideology, organizational structure, and activities changed as a result of closer links with national government. Smith and Lipsky (2009) note for non-profits that closer links with governments can lead, among other things, to more homogeneous services, thus reducing choices for citizens. In addition, Froelich (1999) argues that closer financial ties with the government can lead to a goal shift or the influx of a new clientele. Harris (2010, p. 35) has argued that CSOs ‘find themselves under pressure to change their operating systems and structures so that they complement those of more powerful partners’. This also links to the debate on managerialism in civil society studies (Claeyé and Jackson 2012; Hvenmark 2016; Kreutzer and Jäger 2011; Willner 2019). When management ideology inspired the change towards New Public Management in the public sector, it consequently altered expectations that governments had of CSOs internal operating practices being more business-like, or professional (Anheier 2009).

These studies seem to suggest that CSOs have to give up a part of their dominant organizational features when they get into a service delivery relationship with government. In such a relationship, a *public logic*—or state logic (Thornton et al. 2012)—infiltrates the *community logic* of CSOs, thus resulting in hybrid organizations (Billis 2010; McMullin and Skelcher 2018; Skelcher and Smith 2015). Government–CSO interactions put individual agents for identity struggles, organizational dilemmas, and different options for institutional work. This study provides a closer look at the microprocesses of the relationship and shows how one CSO also guards a community logic as their institutional foundation.

## Theoretical Framework: State–CSO Interactions as Institutional Work

To be able to scrutinize the agentic nature of the government–CSO relationship, an institutional work approach (IWA) is adopted (Lawrence et al. 2013). This approach adds a microinstitutional perspective to the toolkit for institutional analysis. It sheds light on the interplay between institutional environments and the contested meaning over everyday activities, norms, and values within organizations. Thornton et al. (2012) argue that the institutional environment regulates behaviour, as well as creates opportunities for agency and change. When CSOs become involved in public service delivery, they are confronted with a public logic. The IWA draws attention to the embedded agency of individuals in CSOs in the way they organize the tensions between multiple logics (Pache and Santos 2013).

The IWA focuses strongly on the influence of individual actors on the creation, maintenance, and disruption of institutions (Lawrence and Suddaby 2006; Lawrence et al. 2011). How individuals cope with the uncertainty and complex dynamics of plural institutional logics and how they themselves create, maintain, or disrupt such logics are central questions in this body of literature (Lawrence et al. 2011: 55). It adds a microperspective to the toolkit for institutional analysis, bringing everyday ambiguous illustrations of agency into focus (Powell and Rerup 2017). According to Lawrence et al. (2011: 52), this microperspective brings into sight:

a complex mélange of forms of agency—successful and not, simultaneously radical and conservative, strategic and emotional, full of compromises, and rife with unintended consequences. The study of institutional work takes as its point of departure an interest in work—the efforts of individual and collective actors to cope with, keep up with, store up, tear down, tinker with, transform, or create anew the institutional structures within which they live, work, and play, and which give them their roles, relationships, resources, and routines.

In this way, IWA distinguishes itself from a more deterministic macroperspective that puts more emphasis on societal structures rather than agency (DiMaggio and Powell 1983) or a fully agentic approach like rational choice theory. Thornton and Ocasio (2008) argue that ‘the interests, identities, values, and assumptions of individuals and organizations are embedded within prevailing institutional logics. Decisions and outcomes are a result of the interplay between individual agency and institutional structure’ (p.103). Embedded agency presumes the partial autonomy of individuals and organizations within institutional structures.

Institutional work is conducted through intentional actions of collections of individuals (Lawrence and Suddaby 2006; Raviola and Norbäck 2013). The intentions behind institutional work that have been discerned to date are the creation, maintenance, and disruption of institutions (Lawrence and Suddaby 2006; Lawrence et al. 2011). Lawrence and Suddaby (2006) therefore defined institutional work as ‘the purposive action of individuals and organizations aimed at creating, maintaining and disrupting institutions’ (2006: 215). It is because of this intentional and active position of organizational actors that the concept of agency is central to IWA.

Battilana and D’Aunno (2009: 46) define agency as ‘an actor’s engagement with the social world that, through the interplay of habit, imagination and judgement, can both reproduce and transform an environment’s structures’. This definition is based on a multidimensional view of agency by Emirbayer and Mische (1998, in Raviola and Norbäck 2013). They distinguish three dimensions of agency. These are iteration (habit), projection (imagination), and practical evaluation (judgment) (Battilana and D’Aunno 2009; Raviola and Norbäck 2013). *Iteration* focuses on the past and describes the selective reactivation of patterns of meanings and actions by actors, bringing stability and order to the social world and helping to preserve identities, interactions, and institutions through time (Raviola and Norbäck 2013: 1174). The *projective dimension* refers to ‘the imaginative generation by actors of possible future trajectories of action, in which received structures of thought and action may be creatively reconfigured in relation to actors’ hopes, fears and desires for the future’ (Emirbayer and Mische 1998: 971; in Raviola and Norbäck 2013: 1174). The dimension of *practical evaluation* refers to the capacity of actors to respond to emerging dilemmas and ambiguous situations by making a practical and normative assessment of possible alternative courses of action (Battilana and D’Aunno 2009; Raviola and Norbäck 2013).

This three-dimensional view of agency offers a well-developed analytic framework on the actors’ activities and assists in explaining the influence that (coalitions of) actors within CSO scan have in creating, maintaining, or disrupting the institutional relationship with government that is primarily aimed at service provision. Following Battilana and D’Aunno (2009) and Raviola and Norbäck (2013), I apply Emirbayer and Mische’s (1998) conceptualization of agency.

## Methodology

To analyse the ways in which VSCs enact their relationship with government, an organizational ethnographic case study was conducted (Ybema et al. 2009). In an ethnographic study, the researcher develops an informed understanding of the phenomenon being researched through a prolonged presence in the natural environment in which the phenomenon occurs (Denzin and Lincoln 2008). Because of its closeness to the natural environment, organizational ethnography is a strong method in capturing microlevel interactions between government and CSOs. Several authors within the institutional approach stress the importance of more ethnographic research (Lawrence et al. 2013; Thornton et al. 2012), as such research shines light on ‘the mundane, ordinary ways in which institutions are embodied at a micro level and how actors engage with them in their day-to-day activities’ (Lawrence et al. 2013: 1029).

### Case Selection

VSCs are one of those CSOs that are increasingly involved in service delivery. VSCs can thus be viewed as a field-specific example of the broader development of increased state–civil society interactions and their contribution to public value creation (Waardenburg and van de Bovenkamp 2014). The selection of the case was based on the possibility to learn from the case (Flyvbjerg 2006), the proximity of the case for the feasibility of field observations, and the degree of access to information. In advance, the selected case location should: (1) (plan to) participate in a government programme aimed at increasing the wider social role of sports associations; (2) be located in the municipality of Utrecht; and (3) provide the researcher with access to administrative and organizational information such as meetings, minutes, and email conversations.

### Case Study

Until the mid-1990s, sports associations in the Netherlands were primarily regarded as a self-organizing group of people who, together or in competition with others, wanted to experience a sport. Local government funded the build, finance, maintenance, and operation of sport facilities, national governing bodies of sport received institutional grants, and local sport clubs received member subsidies. From the mid-1990s, the national government added a perspective of service delivery, which was subsequently followed by local authorities. Sport was given an important role in public and social challenges such as promoting public health and social integration.

In 2007, national and local governments in the Netherlands made funds available for an impulse to appoint professionals that should form a ‘bridge’ between the educational, sport, and culture sectors to provide children a rich learning environment, in which they could optimize their talents, develop social skills, and have fun. The initial aim was to realize 2.250 fte of community sport coaches (CSC), as the professionals were called. After the CSC had proved itself a promising policy instrument, the policy programme was further developed by the Ministry of Health, Welfare and Sport and continues to this date. In 2020, a total of 3.665 fte has been realized. At the outset, some output goals were: increase the number of primary and secondary schools with sport and cultural activities, especially in deprived areas, and strengthen about 10% of voluntary sport clubs (VSCs) to make them better able to perform their wider social role. The CSC is responsible for organizing after-school sport activities and is often seen as a solution for the absence of organized sport activities in (deprived) neighbourhoods. In 2011, a second round of this program became available for local sport clubs in the municipality of Utrecht.

VVU is an amateur volleyball club in the city of Utrecht, the Netherlands. It was established in 1986 as a result of a merger between two other volleyball clubs. The history of the club dates back to 1945, when volleyball was introduced in the Netherlands by North-American soldiers. VVU is the largest of nine volleyball clubs in Utrecht and one of the three that serves youth volleyball. In the year, I followed the club; it registered 457 members with the National Volleyball Association. The average number of members in volleyball clubs in the Netherlands is one hundred and ten, making the club a relatively large one.

### Data Collection and Analysis

Data were collected by making use of several qualitative methods, such as observations, document analysis, and seventeen semi-structured interviews over a one-year period. I followed the selected VSC for the 2011–2012 season. In this time period, I observed numerous board and committee meetings and two membership meetings, of which I made field notes. My observations further focused on interactive moments between the CSO and government representatives, such as a meeting with a policy advisor. I was able to record most of the meetings. In addition, I had access to the boards e-mail account and Dropbox. I had informal conversations with a range of club members. Finally, I conducted seventeen semi-structured interviews with club representatives in leadership roles, and those members and policymakers directly involved in the process of appointing a CSC at VVU (see Table 1). I invited them to reflect on that process, how they, as individuals were involved in it and how they experienced (parts of) the process. In the following section, I refer to several of these interviewees by using pseudonyms. Interviews on average lasted for seventy-one minutes. All interviews were recorded and transcribed verbatim.

**Table 1:** Overview interviewees

No.	Organization	Role	Male/female	Context
1	Nevobo	Policy advisor	M	Case study
2	Nevobo	Association consultant	M	Case study
3	CSC Support organization	Regional support community sport coaches	F	Case study
4	Utrecht Municipality	Senior policy advisor	F	Case study
5	Local sport support organization	Administrator recreational sport	M	Case study
6	VVU	Board member volunteers	F	Case study
7	VVU	Board member facilities	F	Case study
8	VVU	Community sport coach	F	Case study
9	VVU	Member youth committee	M	Case study
10	VVU	Board treasurer	M	Case study
11	VVU	Board secretary	F	Case study
12	VVU	Technical director	M	Case study
13	VVU	Coach first men’s team	M	Case study
14	VVU	Chairman 1	M	Case study
15	VVU	Chairman 2	M	Case study
16	VVU	Chairman youth committee	M	Case study
17	VVU	Volunteer/former chairman	M	Case study

For the analysis of data, NVivo 10 was used. Due to the diverse sources of information, lump coding was used to manage the amount of data (Saldaña 2009). All interviews and the meetings that emerged from fieldnotes as key moments were transcribed verbatim and coded line by line. On this basis, a chronological case narrative was written. Subsequently, other field notes and information from e-mails and, for example, from club magazines were reviewed in order to identify the connection with and relevance for further interpretation of the constructed case narrative. In view of the focus of this research on microprocesses and agency, the focus of the case studies is on what is said and by whom (Riessman 2008).

The case narrative and the associated data formed the basis for analysis from the institutional work approach. In this analytic phase, I categorized the key moments into the dimensions of agency as discussed in the previous section. Thus, iteration, projection, and practical evaluation, as well as the two central logics—community and public logic—were the most important analytic categories in analyzing the data. I also adopted an open coding strategy. Through this strategy, I was able to recognize a central theme of professionalization and two related themes: competitiveness and managerialism. Jarzabkowski et al. (2009: 309) argue that institutional work takes place with the interaction between actors, with which these actors reproduce or modify existing institutions, create new institutions, and disrupt old ones. The interactions between actors as these emerge in the case narrative illustrate the way in which sports clubs’ members deal with a combination of a community and a public logic. The next section describes and analyses these interactions.

## Findings

This section is structured in accordance with the three-dimensional perspective on agency. The desired ‘professionalization’ of the club is drawn out as a key theme to understand the members’ interpretations of the relationship with and actions towards local government. This theme functions as both a devise for unity and a devise for struggle between some members of the board and members of the youth committee (the most ambitious ‘unit’ of the club). In the final part, I argue that the involvement with the government funded programme has both instrumental and symbolic meanings in light of the clubs’ desired change towards a more professional club. The findings are summarized in Table 2.

**Table 2:** Institutional work

Dimension of agency	Evaluation	Iteration	Projection
Type of institutional work			
Creation	Recognizing the contribution of a CSC to elite sport ambitions and talent development: how can a CSC contribute to professionalization of the club?	Reiteration of the process of professionalization and attention to quality development of coaches	Separate external oriented activities of CSC from internal tasks as coordinator youth coaches
Maintenance	Recognizing the potential of societal activities by means of a CSC: why does this fit us? What does the club get out of it?	Reiteration of growth youth section and attention for quality of coaches as a signifier for the social role the club plays in the community	Incorporate societal projects as a means for professionalization and goodwill
Disruption	Recognizing the dilemma of having the CSC to organize activities in other neighbourhoods: why do we, as a Utrecht-East volleyball club want to organize after school sport activities in Utreht-West?	Reiteration of the importance of focusing on one facility and geographic location for creating a feeling of home and cohesion among members	Start negotiation with municipality over top-down changes in prescribing activities by the CSC

### Iterative Dimension

The iterative dimension of agency sheds light on previous club members’ interpretations and organizational developments. These form a precursor for the projective ideas and practical evaluative actions that will be discussed later.

Throughout its relatively short history (the oldest amateur sport clubs in the Netherlands exist for over 150 years), VVU celebrated some considerable sporting successes. These are memorialized in a 25-year anniversary book (2011) and mentioned several times during post-meeting conversations I had with members of the club. In a similar vein, (inter)national accomplishments of former players or coaches are also singled out. They do not only figure as testimonies of a vibrant history, but also as inspiration for future success. The anniversary book, for instance, contains some articles from a local newspaper from the 1990s and beginning of 2000s, accompanied with a short story explicitly stating: ‘there is the challenge for VV Utrecht to become newsworthy again’. There is a clear wish among volunteers to perform at a higher level in the Dutch volleyball league, especially among those who play(ed) at a high level themselves and/or are involved in talent selection and development.

Board members and a range of volunteers of the club translate this desire as a need for professionalization. The belief in professionalization is widespread among key members. Some years prior to the research period, a committee of members carried out a small study into the feasibility of an association manager for the club. As a result of that study, the general membership meeting (GMM) expressed support for the appointment of an association manager. Although there was support within the club for the appointment of a paid association manager, the association did not poses sufficient funds nor were there subsidies available to realize this. However, from that discussion onwards the issue was always present in the background (interview coach first Men’s team).

However, there are some considerable differences between members in how they interpret what it means to ‘become a professional club’, i.e. what professionalism as an ideology and practice implies for the process of professionalization (see Ganesh and McAllum 2012; Hvenmark 2016). These differences can be traced back to ideas about competitiveness and managerialism, which can be interpreted as two sides of a community logic.

#### Competitiveness

The ambition to become a professional club started when a working group discussed the future of the organization. Eventually, this working group, together with the board of the club, formulated a ‘strategic plan’. In the plan, they formulated a vision for the club:

“The association is a front runner on elite sport and talent development. The possibility to develop talent and ambitions in all segments of the association, makes VVU an alluring association for every volleyball player. With her width and size the association promotes the societal relevance of (volleyball) sport in the area of Utrecht” (strategic plan VVU)

Several further detailed ambitions formulated in the plan regard the development of the youth section in the club, especially talent development. The youth committee seemed to be the most ambitious committee in the club. They had their own strong ideas on how to develop the youth section and the club as a whole. Max, the youth committee’s chairman, a 25-year-old player for the first men’s team and member of the club since he was 11 years old, joined the committee a few years back. In his reasons for taking up this position, he expresses both his discontent and his ambitions:

Max: “I thought it could be better. When I was a youth player myself, little was organized.

I: “What do you exactly mean, ‘little was organized’?

Max: Well, every team had to do it themselves. There was no such thing as policy, we were dressed in old clothes, there were little activities […] Because there were very few teams, players were not really selected.” (interview chairman Youth Committee)

Qualified youth coaches, talent development, and securing youth members for the club, when they make the step to senior teams, are experienced as major issues by the youth committee. Its chair in particular interprets a coherent sport technical policy as a signifier of a professionally managed club, as do others in the organization. Making sure that most youth coaches earn their license with the national volleyball association and technical assistance of these coaches (train the trainer) is one of the youth committee’s major policy goals. So when the possibility of a Community Sport Coach arose, this fitted perfectly in the idea of appointing qualified coaches and training them.

The actions taken by the club’s board were also evaluated in line with sporting experiences and risk-taking, the latter understood as ‘entrepreneurship’:

“Elite sport and entrepreneurship, that was missing gigantically. They just didn’t have a clue. Five thousand Euro was a lot of money in their experience. And it *is* quite a lot of money and you need to be thrifty with it, but if there is a big chance and a small risk that you can earn 2000 Euro by investing those 5000, than you should do that of course.” (Interview chairman Youth Committee)

In the eyes of members focusing primarily on elite sport and talent development, a club needs to make choices to achieve its goals. According to them, this is precisely what was lacking in the last couple of years and is still lacking in decisions made by the board at the time of research. In the end, the board was experienced by some key volunteers as having too little knowledge and experience of what it means and takes to ‘play at a high level’. The reference to experience in high levels of achievement in sport was used as an analogy to express doubts about the ability of leading and organizing the club in such a way that it would be able to perform at a higher level of competition in a more professional way.

#### Managerialism

At the board level, an alternative interpretation of what it means to be a professional club is dominant. Here the emphasis is not on competitiveness and sport technical expertise, but on the stability of the organization. Having a paid employee is in itself experienced as being a more professional club, as it is believed to bolster up the organization. The way the club is managed by the board is also interpreted as a sign of professionalism. Reflecting on his first period in the board, the then Technical Director states:

“Chairman:…now and then it looked like a student board. […] a lot of operational work was talked about. Really, a lot of operational tasks, which are important for that specific person. But those were talked over *in extenso* and that took a long while.

I: Can you give an example?

Chairman: Yes, it could be anything, but the entree fee for a club party or something. […] What I find important is ‘what do we do in five years from now.” (Interview Technical Director/Chairman 2)

Related to this is the way the board tries to create ‘manoeuvre room’ for policy issues and contacts with network partners. This reflects an often mentioned aspect of becoming a more professional club: creating and maintaining a network with external stakeholders. This was felt necessary because without such a network it would be impossible to reach the higher sport level so desired.

The organization is fragmented in several coalitions’ views on what it takes to become a professional club. The main contests in the iterative dimension of agency revolve around diverse meanings of professionalism. One branch sees the skills and quality of coaches, talent development, risk-taking, and ‘daring to make choices’ as symbols of professionalism, the other views strategic planning and financial stability, or managerialism, as indicators for acting professionally.

### Projective Agency: Imagining a Professional Future

Whatever the differences about what it means to become a professional club, all internal actors saw professionalization as a good way to achieve their ambitions. Thus, the iterative dimension provided professionalization as a decision framework for further connections with local government. When the subsidy for a paid employee in the club arose as an opportunity, and it became clear that wages of the employee were fully subsidized, it was quickly decided that this was useful for the club:

“the municipality is encouraged to ensure that a community sport coach is appointed at sports clubs. And that means that such a person could do tasks for our club, but is obliged to spend part of his time on other things. That’s a sort of 60/40 split. 60% is allowed within the association and 40% must then focus on other things. And in our case, for example, schools would be a very good target group for that, to put energy on it. Uhm… given the discussion we had in the past about the club manager and the desire within the association to professionalize in that area, we as the board thought that this was a good opportunity for us. Especially because at the time of the discussion we said ‘yes, we would like to, but we have to make sure that there is also funding for that. Because someone who gets paid is really a completely different cost structure than what you normally have in the club’. And in the case of a CSC, it’s actually the case that such a person can start working fully subsidised. So uh… in that light we decided to make an application eh… to have someone like that start on January 1st. “ (GMM, 29-10-2011)

In the second part of the quote, the chairman links the policy impulse with terms such as ‘club manager’ and ‘professionalization’. These terms are not foreign to the members present at the GMM. In the excerpt above, the chairman mentions professionalization of the association as the part of the appointment of the CSC that concerns the ‘internal aspect for the association’. In this way the chairman identifies professionalization and connecting with external organizations as two separate responsibilities and organizational processes. At the end of the quote, the chairman refers to the financial construction of the CSC. The fully subsidized professional prompted the board to apply for a CSC. Thus, the CSC as a fully subsidized club manager who contributes to the professionalization of the club emerges as the dominant meaning of the CSC in the chairman’s explanation at the general membership meeting. The attendees do not ask any further questions on this subject and accept and support this projective interpretation of the policy impulse.

### Practical Evaluative Dimension: Judging the Interaction

During a meeting with a senior administrator from the municipality, the wish to use the CSC also for managerial tasks is discussed. This administrator informed all clubs who were granted the subsidy about the specifics of the subsidy. When the chairman talks about coordinating tasks, the administrator responds ‘the Community Sport Coach should specially be used at the work floor’ (meeting, 28-11-2011). With this statement, the municipal administrator underscores the policy desires expressed in the ministerial agreements of the program: ‘the impulse will not be used to fund existing positions. As much as possible will be allocated to positions on operational level.’ (OCW and VWS 2007)

The chairman responds that they are willing to reformulate some parts of the job function. In the end, the CSC has not become responsible for coordinating volunteers indeed. However, she does have some coordinating tasks, for instance, coordinating youth trainers in the youngest youth section of the club.

Six months after the appointment of the CSC, the municipality decided to intervene in the tasks of the CSC. The board and those closely cooperating with the CSC were ‘shocked’. After an initial period of relative freedom for the club in assigning tasks to the CSC, the municipality decided to rotate all appointed CSCs throughout the city. It does this mainly to promote sports participation in all neighbourhoods and to ensure a diverse range of sports in those areas. This meant the CSC had to organize after school volleyball activities in areas at the other side of town. This is aneighbourhood where only a marginal percentage of members of the club come from and where no other volleyball clubs that have a youth section are active. The municipality wanted volleyball activities aimed at youth in all parts of the city, while VVU saw more relevance in delivering activities in the central and Eastern neighbourhoods where youth would actually have a further possibility in joining the club. The CSC herself underscores the clubs’ perspective:

“In 8 weeks we will be assigned to Leidsche Rijn. And we consider that to be totally useless, because… We can give clinics there, but those children don’t come here, that’s too far away. The clubs over there don’t enlist youth anymore, so that just doesn’t make any sense.” (interview CSC)

Reacting to this top-down change, the chairman tries to persuade the municipal contact person to revise the municipalities’ position on the prescribed activities for the CSC. He is unsuccessful in his endeavor.

Through the change in the policy relationship, the municipality uses the CSCs as a backdoor to redirect the societal activities of the club more towards public purposes. This is in line with the agreements made at the start of the relationship. In the end, the CSC starts delivering activities in the other side of the city. Finding a balance between and committing to the public and club-oriented activities proves to be an unexpected and important challenge for the CSC and her voluntary employer. Once again this points to the dominant meaning that those involved give to the CSC. Their attention is not focused on the external service provision by the CSC, but on the value this function has for the professionalization of the club.

## Discussion

This article aimed to explore the microfoundations of government–CSO interactions. Through the institutional work approach (Lawrence et al. 2011, 2013), the analysis makes a connection between the macrolevel of institutional logics, and the microlevel interplay between CSO agents and local government. The Dutch CSC programme was used as a case to illustrate the usefulness of the IWA for analysing these microfoundations. The case analysis provides several important lessons about government–CSO interactions.

Through a three-dimensional perspective on agency (Emirbayer and Mische 1998), it was possible to identify how individual agents enacted the interplay between different institutional logics. In the selected case, a community logic is prevalent. The selected CSO contributes to the CSC programme, primarily from a community logic that focusses on competitiveness and managerialism (cf. Meyer et al. 2013; Skille 2011; Stenling and Fahlén 2009). This can be viewed as prospective agency, hoping to boost its image as a professionally run sport club, to gain field level legitimacy and goodwill with the municipality (Meyer et al. 2013). The case shows an assimilation of elements of the public logic into the community logic. Members of the studied sport club only sparsely connect with such aims as increasing the overall sport participation in a specific neighbourhood or improving the social cohesion in that area. Furthermore, the VSC does not approach its surrounding environment in terms of public target groups, like immigrants or the elderly. Whatever their internal differences, what the club members shared was an instrumental motivation towards public policy objectives and an absence of intrinsic motivations for what one could call a wider social role for sport clubs (Waardenburg 2016). Such differences facilitate the resistance—or disruptive institutional work—towards a public logic as well as the assimilation of public policy objectives into the dominant community logic. This finding is in line with existing literature on VSCs as policy implementers, highlighting an implementation challenge due to the competitive orientation of most VSCs (Ibsen and Levinsen 2019; Skille 2011; Stenling and Fahlén 2009; Waardenburg and Nagel 2019).

Another observation is that this form of assimilation of a public logic into a community logic also presumes institutional change (Skelcher and Smith 2015; Thornton et al. 2012); in the end, service delivery has been assimilated as a symbol and as a practice into the sport clubs’ dominant community logic. Service provision has by now become part of the vocabularies and the activities of many CSOs (Brandsen and Pestoff 2006; McMullin and Skelcher 2018). From an institutional work approach, this can be interpreted as practice work; change occurs in the legitimized practices of CSOs. Through practice work, the studied CSO assimilates elements of a public logic, thereby *maintaining* a community logic. These findings correspond with the observation that plural logics can coexist alongside each other (Besharov and Smith 2014; Kraatz and Block 2008; Skelcher and Smith 2015; Skirstad and Chelladurai 2011). The microfoundational perspective adds to this the observation that individual agents, through a multivocality of voices, actively engage with these plural logics (see also Felder et al. 2018).

The application of IWA also highlights the double-sided relation between service provision and managerialism. Previous studies predominantly describe this relationship as unidirectional. When CSOs become involved in service delivery, expectations about their managerial ability rise. Thus, managerialism has often been addressed as a consequence of adopting a role as service provider by CSOs (Cairns et al. 2005; Harris 2010). The case analysis shows another possible connection that managerialism in a CSO triggers the openness to and adoption of a service delivery role. An important reason for CSOs to cooperate with government for service provision is securing strategic resources and increasing goodwill (Sowa 2009; Willner 2019). While the studied CSO is not financially dependent on the municipality, members evaluate subsidies as a possibility to finance the professionalization of their association. Government subsidies are perceived as an alternative financial model to realize the appointment of a club manager. Governments and commercial activities increasingly provide possibilities for financing the professionalization of VSCs. Studies into other types of CSOs show that the professionalization of the association through paid professionals could put VSCs for new financial and other organizational challenges (Billis 2010; Cairns et al. 2005). According to Billis (2010), taking on the first paid staff should be seen as a form of shallow hybridity. Often such modest hybridity arises from the desire to extend the range of activities. Public instruments and activities thus form an instrument for CSOs to position themselves vis-à-vis external stakeholders. The findings of this study suggest that the agentic position towards this public logic delineates the specific form (e.g. segregating, assimilating, blending) the process of hybridization takes (see also McMullin and Skelcher 2018).

Finally, seeking cooperation with state actors bears the risk of government co-optation (Najam 2000). This case, however, is better understood as an instance of mutual manipulation, in which both parties use each other to achieve their own ends. The institutional work approach, and its three-dimensional perspective of agency, assisted in exploring and describing this dual nature of the relationship.

There are some limitations to this study. First, this study provides what has been dubbed analytic generalizable insights, not empirically generalizable results (Thomas and Myers 2015). Thus, what was observed in the case study cannot readily be transferred to other sports clubs or CSOs without careful consideration of those organizational contexts. A second limitation is that the case revolves around a specific policy instrument, the Community Sport Coach. As such, the case study was limited to those persons that were most directly involved with deciding on and implementing this policy instrument and related services, and not the whole membership base of the club. This may have decreased the possibility of generating a broad range of experiences and could have added another layer of understanding why professionalization, competitiveness, and managerialism are such strong forces in the organization. Another limitation of the study is the context of the voluntary sport sector, with its relatively strong focus on competitiveness. This field-specific dominant logic in sports could mean that other civil society sectors might be less able to maintain a community logic. This is something up for further research.

Follow-up research could thus focus attention on other civil society sectors. In what ways do CSOs in e.g. human services, health, or religious sectors enact their interactions with government at a microlevel? And what does institutional work entail for this relationship when focusing our attention to more authoritative regimes (e.g. Skokova et al. 2018) or advocacy-oriented CSOs? Such research might be based on similar in depth single case studies, multiple single sector case studies, or comparative multiple sector case studies. All types of case study design have the ability to tighten our grip on the microfoundations of government–civil society interactions. Another approach could consist of qualitative longitudinal research (e.g. Chen 2009). Whilst the chosen timeframe for this study provides sufficient depth for our current conclusions, it remains to be seen how the studied relationship further develops, especially when public policy objectives and/or instruments change over time. This could provide a better understanding which policy instruments are considered more impactful for CSOs and how they enable or disable their microlevel endeavours in institutional work.

## Conclusion

In short, this article concludes that CSOs (can) actively enact their relationship with local government and in the process maintain a community logic. Members of the organization under study initially respond positively to government’s call for service provision, in line with a previously iterated desire for professionalization. Members see in the CSC programme a projective opportunity for the development of their own organization. They are willing to commit themselves to a broader clientele in exchange for public funds that help them to achieve their competitive ambitions and run the organization in a more managerial fashion. CSOs certainly are no passive bystander in the service delivery relationship. By assimilating elements of a public logic, the case organization institutionally maintains its community logic. The study illustrated the usefulness of the institutional work approach in further understanding government–CSOs interactions and dissect the multivocality of these interactions.
